# Clinical relevance of aortic calcification in urolithiasis patients

**DOI:** 10.1186/s12894-017-0218-2

**Published:** 2017-04-04

**Authors:** Toshikazu Tanaka, Shingo Hatakeyama, Hayato Yamamoto, Takuma Narita, Itsuto Hamano, Teppei Matsumoto, Osamu Soma, Yuki Tobisawa, Tohru Yoneyama, Takahiro Yoneyama, Yasuhiro Hashimoto, Takuya Koie, Ippei Takahashi, Shigeyuki Nakaji, Yuriko Terayama, Tomihisa Funyu, Chikara Ohyama

**Affiliations:** 1 Department of Urology, Hirosaki University Graduate School of Medicine, 5 Zaifu-chou, Hirosaki, 036-8562 Japan; 2 Department of Advanced Transplant and Regenerative Medicine, Hirosaki University Graduate School of Medicine, Hirosaki, Japan; 3 Department of Urology, Oyokyo Kidney Research Institute, Hirosaki, Japan; 4 Department of Social Medicine, Hirosaki University School of Medicine, Hirosaki, Japan

**Keywords:** Urolithiasis, Stone former, Aortic calcification, Chronic kidney disease, Renal function

## Abstract

**Background:**

The aim of the present study is to investigate the clinical relevance of aortic calcification in urolithiasis patients.

**Methods:**

Between January 2010 and September 2014, 1221 patients with urolithiasis were treated in Oyokyo Kidney Research Institute and Hirosaki University Hospital. Among these, 287 patients (Stone group) on whom adequate data were available were included in this retrospective study. We also selected 148 subjects with early stage (pT1N0M0) renal cell carcinoma from 607 renal cell carcinoma patients who underwent radical nephrectomy at Hirosaki University Hospital (Non-stone group) as control subjects. Validity of the Non-stone group was evaluated by comparison with pair-matched 296 volunteers from 1166 subjects who participated in the Iwaki Health Promotion Project in 2014. Thereafter, age, body mass index, aortic calcification index (ACI), renal function, serum uric acid concentrations, and comorbidities (diabetes, hypertension, or cardiovascular disease) were compared between the Non-stone and Stone groups. Independent factors for higher ACI and impaired renal function were assessed using multivariate logistic regression analysis.

**Results:**

We confirmed relevance of Non-stone group patients as a control subject by comparing the pair-matched community-dwelling volunteers. Backgrounds of patients between the Non-stone and Stone groups were not significantly different except for the presence of hypertension in the Stone group. ACI was not significantly high in the Stone group compared with the Non-stone group. However, age-adjusted ACI was greater in the Stone group than the Non-stone group. Among urolithiasis patients, ACI was significantly higher in uric acid containing stone patients. The number of patients with stage 3B chronic kidney disease (CKD) was significantly higher in the Stone group than in the Non-stone group (12% vs. 4%, *P* = 0.008). Multivariate logistic regression analysis showed higher aortic calcification index (>13%), and being a stone former were independent factors for stage 3B CKD at the time of diagnosis.

**Conclusion:**

Aortic calcification and being a stone former had harmful influence on renal function. This study was registered as a clinical trial: UMIN: UMIN000022962.

## Background

Urolithiasis is a common urological disease, and its prevalence has been increasing in Japan similar to that in other developed countries [1]. In 2005, in Japan, the age-standardized annual incidences of the first episode of upper tract stones were reported to be 165.1/100,000 men and 65.1/100,000 women, which were 2-fold higher compared with that in 1965 [2]. During this period, lifestyle and dietary habits in Japan were more westernized, and the prevalence of obesity and metabolic syndrome (MetS) increased rapidly [3]. MetS refers to a cluster of risk factors, including high blood pressure, obesity, high cholesterol, type 2 diabetes, and atherosclerotic cardiovascular disease [4]. Moreover, many epidemiological studies have suggested an association among urolithiasis, MetS, and chronic kidney disease (CKD) [5–7]. Because urolithiasis is considered as one of the consequences of MetS, we hypothesized that concurrent aortic calcification, complicated by arterial stiffness and atherosclerosis, may be responsible for developing CKD. Aortic calcification has recently been considered as a major complication and an independent risk factor for CKD, coronary diseases, heart failure, and stroke [8–10]. Aortic calcification is widely used as an indicator of MetS-related disease [11], and it can be quantitatively measured by the aortic calcification index (ACI) using abdominal computed tomography (CT). We have previously reported the clinical relevance of ACI in hemodialysis patients, renal transplant recipients, and primary aldosteronism patients [12–14]. However, only a few studies have investigated aortic calcification and urolithiasis [15], and the implication of aortic calcification in urolithiasis patients remains unclear. In the present study, we retrospectively assessed the distribution of aortic calcification, and influence of aortic calcification on renal function in urolithiasis patients. This study was registered as a clinical trial: UMIN000022962.

## Methods

**Fig. 1:**
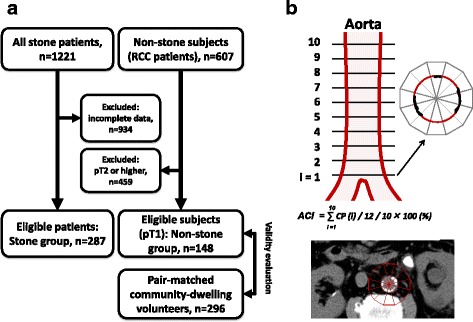
Patient selection and measurement of aortic calcification index. Eligible stone patients and non-stone subjects were selected from our database in Oyokyo Kidney Research Institute and Hirosaki University Hospital. In the Stone group, 934 patients were excluded because of incomplete data. The non-stone subjects were selected from renal cell carcinoma (RCC) patients who underwent radical nephrectomy at Hirosaki University Hospital. Of those, we selected early-stage RCC patients (pT1N0M0) for the non-stone control subjects (Non-stone group). Validity of the Non-stone group was evaluated by comparison with pair-matched 296 community-dwelling volunteers from 1166 subjects who participated in the Iwaki Health Promotion Project in 2014 (**a**). Aortic calcification was quantitatively measured using pretreatment abdominal computed tomography images, scanned 10 times at 10-mm intervals above the abdominal aortic bifurcation. The calcification profile was calculated as the sum of calcification areas of 12 fractions in a single slice divided by 12. The sum of the calcification profile from 10 slices was divided by 10 and multiplied by 100 to obtain the percentage The typical computed tomography (CT) of stone patient is shown. The aortic calcification index (ACI) of this section is 10/12 × 100 = 83.3% (**b**)

Between January 2010 and September 2014, we treated 1221 stone patients with urolithiasis in Oyokyo Kidney Research Institute and Hirosaki University Hospital. We excluded the patients whose data including stone information and blood exam were inadequate. As a result, the remaining 287 patients (Stone group) who underwent pre-treatment abdominal CT and laboratory testing such as renal function, uric acid, lipid metabolism, and urinalysis were included in this retrospective study (Fig. 1a). The non-stone subjects comprised renal cell carcinoma (RCC) patients who had undergone radical nephrectomy at Hirosaki University Hospital between December 1986 and March 2015. Because pre-treatment abdominal CT and laboratory testing were necessary in those patients, we selected early stage RCC patients as a control group. Of 607 RCC patients, 148 early stage RCC patients (pT1N0M0) were selected as the non-stone control subjects (Non-stone group). Laboratory testing was performed before stone treatment or radical nephrectomy. The estimated glomerular filtration rate (eGFR) was calculated using the Modification of Diet in Renal Disease equation for Japanese patients [16].

To guarantee the validity of early stage RCC patients, we employed pair-matched 296 community-dwelling volunteers from 1166 subjects who participated in the Iwaki Health Promotion Project in 2014 [17]. The demographic data (age, sex, body mass index) and medical information (positive history of hypertension, cardiovascular disease, diabetes, and dyslipidemia) were obtained from self-questionnaires and interviews in this project. To select appropriate subjects, we used the propensity score matching strategy to compare renal function as described previously [18]. Propensity scores were calculated using logistic analysis, and the data used in the analyses included age, gender, body mass index, positive history of cardiovascular disease, hypertension, and diabetes. Based on the scores, two healthy subjects and one RCC patients with a score within 0.03 were selected as a pair (at a 2:1 ratio), and we compared renal function, serum uric acid concentration and lipid disorder between the volunteers and Non-stone group.

In the Non-stone and Stone groups, ACI was quantitatively measured using abdominal CT images (TSX-301B, Toshiba Medical Systems Corp., Ohtawara, Japan, or CT750HD, GE Healthcare Japan, Tokyo, Japan) above the common iliac artery bifurcation by scanning 10 times at 10-mm intervals, as described previously [12]. ACI (%) expresses the calcification proportion in 12 sectors, and it is calculated as the average value of sections 1–10 (Fig. 1b).

After the validity evaluation of early stage RCC patients for control subjects, we investigated the distribution of ACI in urolithiasis patients. Patients’ background including age, sex, body mass index, comorbidities (diabetes, hypertension, or cardiovascular disease), renal function, lipid metabolism, serum uric acid concentrations, voluntary urine protein, and ACI were compared between the Non-stone and Stone groups. Thereafter, the impact of stone disease on renal function were compared between the Non-stone and Stone groups.

### Statistical analysis

The statistical analyses of the clinical data were performed using SPSS v. 22.0 (IBM Corporation, Armonk, NY, USA) and GraphPad Prism v. 5.03 (GraphPad Software, San Diego, CA, USA). Categorical variables were reported as percentages and compared using Fisher’s exact test or Chi-square test. Quantitative data were expressed as medians with quartiles 1 and 3 (Q1-Q3). Differences between the groups were statistically compared using Student’s *t*-test for data with normal distribution or Mann–Whitney *U*-test for data exhibiting a non-normal distribution. Differences among the three groups were statistically compared using Kruskal-Wallis test. When significant differences among the three groups were observed, we performed multiple comparisons. The correlation between two indices was analyzed using Spearman’s correlation coefficient. Probability (*P*) values of <0.05 were considered to be statistically significant. The optimal cut-off value for stage 3B CKD was calculated using the following formula [19] :(1 − sensitivity)^2^ + (1 − specificity)^2^ with the receiver operating characteristic (ROC) curve.

Independent factors influencing stage 3B CKD at the time of diagnosis were identified by multivariate analyses using a logistic regression model. Odds ratios (ORs) with 95% confidence intervals (CIs) were calculated after concurrently adjusting for potential confounders. Data from healthy volunteers were not included in this multivariate model due to the absence of ACI and proteinuria information. We included nine variables in the logistic regression analysis because of the limited number of samples. Several independent variables that increased the postoperative renal impairment risk were included in the models: age (>65 years), sex (male), history of comorbidity (hypertension, type 2 diabetes, or cardiovascular disease), lipid metabolism abnormality (total cholesterol > 220 mg/dL or triglyceride > 140 mg/dL), serum uric acid concentration (>7.0 mg/dL), voluntary urine protein (>30 mg/dL), and ACI (>13.0%) at the time of diagnosis. Moreover, the predictive accuracy of the selected variables in the dataset was evaluated using the area under the curve (AUC) derived from the ROC curve.

## Results

### Comparison between community-dwelling volunteers and early stage RCC patients

We compared pair-matched 296 community-dwelling volunteers and 148 of early stage RCC patients. Median age, sex, body mass index, positive history of hypertension, diabetes, and cardiovascular disease were not significantly different. In addition, unadjusted parameters such as eGFR, the number of subjects with stage 3B CKD, hyperuricemia, and dyslipidemia were also not significantly different (Table 1). Based on these results, we regarded early stage RCC patients as appropriate candidate for control subjects.

**Table 1:** Clinical characteristic of community-dwelling volunteers and early stage renal cell carcinoma patients (Non-stone group)

	Volunteers	Non-stone group	*P value*
n	296	148	
Age (years)^a^	64 (56–72)	62 (54–72)	*0.333*
Gender (Male)^a^, *n=*	176 (59%)	89 (60%)	*0.919*
Body mass index^a^ (kg/m^2^)	24 ± 4	24 ± 4	*0.849*
Comorbidities			
Hypertension^a^, *n=*	85 (29%)	43 (29%)	*1.000*
Diabetes^a^, *n=*	47 (16%)	31 (21%)	*0.189*
Cardiovascular disease^a^, *n=*	42 (14%)	20 (14%)	*0.886*
eGFR (mL/min/1.73 m^2^)	76 (66–85)	72 (61–87)	*0.127*
Stage 3B CKD, *n=*	12 (4%)	6 (4%)	*1.000*
Uric acid > 7.0 mg/dL, *n=*	34 (11%)	24 (16%)	*0.180*
Dyslipidemia (Total cholesterol >220, or Triglyceride >140 mg/dL), *n=*	21 (7%)	15 (10%)	*0.401*
Urine protein > 30 mg/dL, *n=*	N/A	22 (15%)	
Aortic calcification index (ACI)	N/A	6.7 (0.8-19.2)	

Median and interquartile range (Q1-Q3) was used for consecutive variables

^a^, applied for propensity score-matching

### Comparison between early stage RCC patients (Non-stone group) and stone patients (Stone group)

**Fig. 2:**
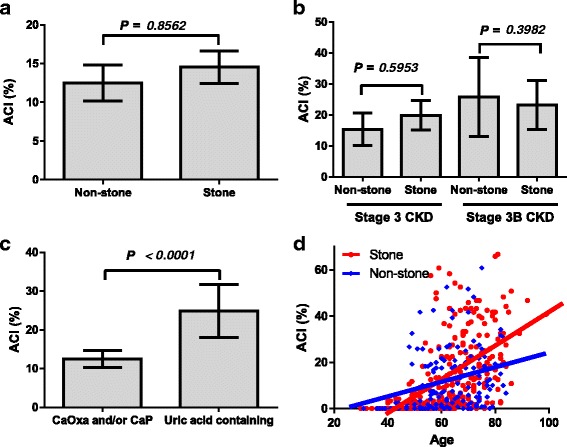
Aortic calcification index (ACI) between control subjects and stone patients. ACI was not significantly different between the Non-stone and Stone groups (**a**). In the stage 3 or 3B CKD patients, there are no significant differences in ACI between the groups (**b**). ACI was significantly higher in uric acid containing stone patients (**c**). Scatter plot analyses are performed to compare the relationship between aortic calcification index (ACI) and age. Linear approximations of ACI and age show a positive correlation in the Non-stone (*blue line*, R^2^ = 0.071, *P* <0.001) and Stone group (*red line*, R^2^ = 0.285, *P* < 0.001) (Spearman’s correlation coefficient test). Age-adjusted ACI (a slope of line) is greater in the Stone group (0.744) compared with the Non-stone group (0.468) (**d**)

Thereafter, we compared the Non-stone and Stone groups. Component of stones were calcium oxalate (*n* = 119, 41.5%), calcium oxalate mixed stones (*n* = 97, 33.8%), uric acid containing stone (*n* = 31, 10.8%), cysteine (*n* = 1, 0.35%), and unknown (*n* = 39, 13.6%). Table 2 summarizes the patient characteristics. The number of patients with hypertension was significantly higher in the Stone group (*P* = 0.001). No significant differences were observed for any other parameter. ACI was not significantly different between the groups (Fig. 2a). In addition, there were no statistical significant differences in ACI in patients with stage 3 CKD or stage 3B CKD (Fig. 2b). Among urolithiasis patients, ACI was significantly higher in uric acid containing stone patients (Fig. 2c). Age and ACI showed positive correlations in the Stone group (R^2^ = 0.285, *P* < 0.001) and Non-stone group (R^2^ = 0.071, *P* < 0.001, Spearman’s correlation coefficient test). Age-adjusted ACI (a slope of the line) was greater in the Stone group (0.744) than the Non-stone group (0.468) (Fig. 2d).

**Table 2:** Patients’ characteristics

	Non-stone group	Stone group	*P value*
n	148	292	
Age (years)	62 (54–72)	63 (54–72)	*0.626*
Sex (Male), *n=*	89 (60%)	169 (58%)	*0.683*
Body mass index (kg/m^2^)	24 ± 4	25 ± 4	*0.171*
Comorbidities			
Hypertension, *n=*	43 (29%)	142 (49%)	*<0.001*
Diabetes, *n=*	31 (21%)	72 (25%)	*0.407*
Cardiovascular disease, *n=*	20 (14%)	33 (11%)	*0.536*
eGFR (ml/min/1.73 m^2^)	72 (61–87)	76 (58–95)	*0.250*
Hyperuricemia (>7.0 mg/dL, *n=*	24 (16%)	45 (15%)	*0.890*
Dyslipidemia (total cholesterol >220, or triglyceride >140 mg/dL), *n=*	15 (10%)	31 (11%)	*1.000*
Voluntary urine protein > 30 mg/dL, *n=*	22 (15%)	57 (20%)	*0.240*
Type of stone			
Uric acid stone, *n=*		32 (11%)	
Non-uric acid stone, *n=*		216 (74%)	
Unknown, *n=*		44 (15%)	
Aortic calcification index (ACI)	6.7 (0.8–19.2)	7.1 (0.8–22.5)	*0.856*

Median and interquartile range (Q1, Q3) was used for consecutive variables

**Fig. 3:**
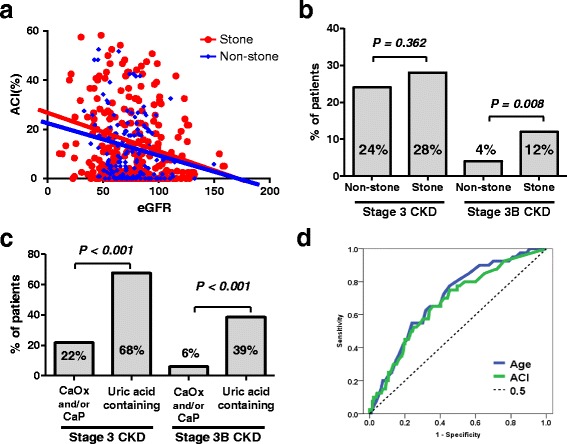
Correlation eGFR and aortic calcification index (ACI), chronic kidney disease (CKD), and receiver operating characteristic curve (ROC) analysis for predictive accuracy of stage 3B chronic kidney disease. ACI and eGFR showed significant, but weak correlations in the Stone (R^2^ = 0.053, *P* < 0.001) and Non-stone group (R^2^ = 0.032, *P* = 0.029) (**a**). The number of patients with stage 3B CKD was significantly higher in the Stone group compared with the Non-stone group (12% vs. 4%, *P* = 0.008), although the number of patients with stage 3 CKD was not significantly different (**b**). The number of patients with stage 3 and 3B CKD was significantly higher in uric stone containing patients compared with calcium oxalate (CaOx) and/or calcium phosphate (CaP) stone patients (**c**). The optimal ACI cut-off value of age and ACI for stage 3B CKD was determined by analyzing ROC *curves* using the area under the *curve* (AUC) (**d**). An age of 65 years (AUC = 0.70; *P* < 0.001; 95% CI: 0.62–0.78, *blue line*) and ACI of 13.0% (AUC = 0.68; *P* < 0.001; 95% CI: 0.59–0.76, *green line*) were used as the cut-off values in this study

Although ACI and eGFR showed negative correlations (Fig. 3a), R^2^ values showed weak correlation in both groups (Stone: R^2^ = 0.053, *P* < 0.001, and Non-stone: R^2^ = 0.032, *P* = 0.029). The number of patients with stage 3 CKD was not significantly different between the Non-stone and Stone groups (28% vs. 24%, *P* = 0.362). However, the number of patients with stage 3B CKD was significantly higher in the Stone group compared with the Non-stone group (12% vs. 4%, respectively, *P* = 0.008) (Fig. 3b). Similarly, patients with uric acid containing stone were higher in stage 3A/3B CKD (Fig. 3c, Table 3).

**Table 3:** Relationship between stage of CKD and stone components

	Uric acid (%)	CaOx / CaP (%)	*P value*
Normal / CKD 1	0 (0%)	70 (32%)	*<0.001*
CKD 2	9 (41%)	99 (46%)	*0.078*
CKD 3A	9 (41%)	34 (16%)	*0.068*
CKD 3B	5 (19%)	10 (5%)	*0.027*
CKD 4	7 (29%)	3 (1%)	*<0.001*

*CaOx* calcium oxalate, *CaP* Calcium phosphate

To investigate the implications of ACI in renal function, the optimal ACI cut-off value for age and ACI was determined by analyzing ROC curves. An age of 65 years (AUC = 0.70, *P* < 0.001, 95% CI: 0.62–0.78) and ACI of 13.0% (AUC = 0.68, *P* < 0.001, 95% CI: 0.59–0.76) were used as the cut-off values in this study (Fig. 3d).

**Fig. 4:**
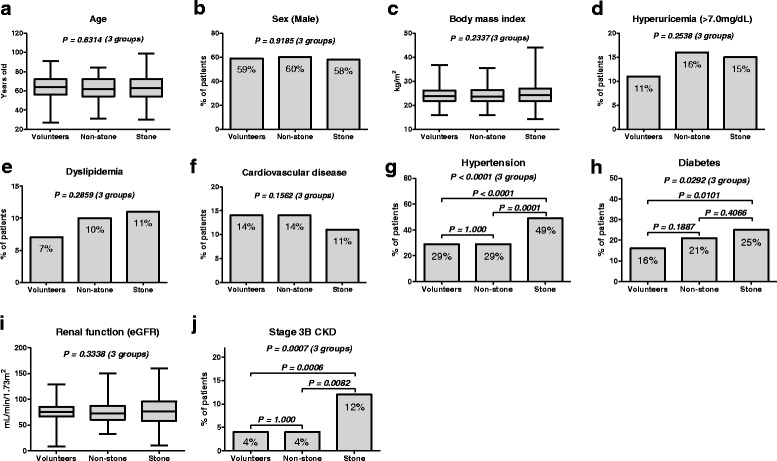
The three group comparisons among the community-dwelling volunteers, Non-stone, and Stone groups. There were no significantly differences in age (**a**), sex (**b**), body mass index (**c**), hyperuricemia (**d**), dyslipidemia (**e**), positive history of cardiovascular disease (**f**) and eGFR (**i**), except for hypertension (**g**), diabetes (**h**), and prevalence of stage 3B CKD (**j**). It is remarkable that the prevalence of stage 3B CKD is significantly higher in the Stone group

Multivariate logistic regression analysis revealed that an age of >65 years, sex (male), presence of comorbidities, serum uric acid (>7.0 mg/dL) were selected as independent factors for higher ACI. Similarly, an age of >65 years, sex (male), hyperuricemia, voluntary urine protein > 30 mg/d, ACI >13.0%, and Stone group were selected as independent factors for stage 3B CKD at the time of diagnosis (Table 4). The three group comparisons among the community-dwelling volunteers, Non-stone and Stone groups were shown in Fig. 4.

**Table 4:** Multivariate logistic regression analyses of independent factors for higher ACI (>13%) and stage 3B CKD or higher (eGFR < 45 mL/min/1.73 m^2^) at the time of diagnosis between the Non-stone and Stone groups

ACI	Factors	*P value*	OR	95% CI
Age	> 65 years	*0.000*	3.90	2.47-6.17
Sex	Male	*0.013*	1.76	1.13-2.76
Body mass index	> 25 kg/m^2^	*0.132*	0.71	0.46-1.11
Comorbidities	Positive	*0.049*	1.60	1.00-2.55
Lipid metabolism abnormality	Positive	*0.337*	0.80	0.51-1.26
Serum uric acid	> 7.0 mg/dL	*0.047*	0.52	0.27-0.99
Urine protein	> 30 mg/dL	*0.089*	1.63	0.93-2.87
CKD stage	3B or higher	*0.052*	2.17	0.99-4.74
Stone formers	Positive	*0.501*	0.85	0.54-1.35
Stage 3B CKD	Factors	*P value*	OR	95% CI
Age	> 65 years	*0.004*	4.11	1.59-10.6
Sex	Male	*0.030*	2.56	1.09-5.99
Body mass index	> 25 kg/m^2^	*0.714*	1.15	0.54-2.44
Comorbidities	Positive	*0.606*	1.26	0.52-3.03
Lipid metabolism abnormality	Positive	*0.904*	1.05	0.49-2.27
Serum uric acid	> 7.0 mg/dL	*0.001*	4.53	1.94-10.6
Urine protein	> 30 mg/dL	*0.003*	3.22	1.50-6.91
ACI	> 13.0%	*0.047*	2.28	1.01-5.17
Stone formers	Positive	*0.005*	4.00	1.52-10.5

Comorbidities included history of diabetes, hypertension, or cardiovascular disease

*ACI* aortic calcification index

## Discussion

Aortic calcification is regarded as one of the consequences of systemic aortic degradation. Numerous studies have addressed the clinical importance of arterial calcification among CKD patients and cardiovascular high-risk patients [10–12, 14, 19–21]. Our previous study suggested that aortic calcification burden has a negative effect on postoperative renal function in renal transplant patients, [14] and the preoperative condition of aortic calcification has a significant impact on postoperative persistent hypertension after unilateral adrenalectomy in patients with aldosterone-producing adenomas. [13] However, only a few studies have demonstrated the impact of arterial calcification in urolithiasis patients. Yasui et al. [15] reported that aortic calcification scores were significantly different between stone and non-stone patients. They also suggested that a diet with high animal protein, cholesterol, and fat may cause urolithiasis and arteriosclerosis. Therefore, we hypothesized that aortic calcification may play an important role in renal function deterioration in urolithiasis patients. However, our results suggested that ACI was not significantly higher in urolithiasis patients with CKD, whereas the age-related aortic calcification is greater in urolithiasis patients. Although we could not prove the relationship between ACI and CKD in urolithiasis patients, the number of stage 3B CKD patients was significantly higher in the Stone group. The prevalence of stage 3B CKD in the community-dwelling volunteers, Non-stone, and Stone group showed 4%, 4%, and 12%, respectively (Fig. 4J). In addition, multivariate analysis identified ACI and being a stone former as potential factor for stage 3B CKD, which is similar to age, gender, hyperuricemia, and proteinuria. Our finding suggested that patients with poor renal function have more aortic calcification in stone formers as well as the control subjects. On the other hand, being a stone former was not selected as independent factors for higher ACI, whereas age, sex and hyperuricemia were associated with higher ACI. These results suggest a complicated relationship among vascular calcification, CKD and stone formation. Although precise mechanisms for urinary stone formation and aortic calcification remain unclear, higher age-adjusted ACI in urolithiasis patients suggested that MetS, including obesity, hypertension, impaired glucose metabolism, hyperuricemia, and atherogenic dyslipidemia, may play a key role in the vascular calcification and urolithiasis development, and resulted in premature vascular aging in stone formers.

Although mechanisms responsible for vascular calcification is still under investigation, it has been reported that vascular smooth muscle cells play a critical role in mediating vessel calcification by differentiating into osteoblast-like cells [22]. Calcifying vascular cells, which are a subpopulation of vascular smooth muscle cells, spontaneously form ossified nodules when cultured for a long time. These nodules express many bone-related molecules, including increased alkaline phosphatase activity, and osteocalcin, osteonectin, and osteopontin expressions [23]. Osteopontin is a glycoprotein secreted by macrophages, vascular smooth muscle cells, and endothelial cells and has been demonstrated to promote macrophage chemotaxis [24, 25]. It has been identified as a major matrix component of urinary calcium stones [26] and is strongly associated with urinary stone formation [27]. Recent studies have suggested that renal tubular inflammation, involving macrophages and osteopontin, plays a key role in renal calcium oxalate crystal formation [28]. Inflammation and osteopontin also play key roles in vascular calcification [25, 29] and inflammation, impaired calcium and phosphate homeostasis, and oxidative stress have been linked to vascular calcification in CKD patients. [30]. Furthermore, MetS has been considered as a chronic, low grade, systemic inflammatory disease [31]. Based on these results, vascular calcification and urinary stone formation might share similar mechanisms through inflammation, which is regarded as the key process underlying metabolic diseases. Further studies are necessary to address the detailed association between MetS, inflammation, oxidative stress, vascular calcification, urinary stone formation, and CKD.

Several limitations in this study need to be noted. The small sample size and retrospective design prevented definitive conclusions on the aortic calcification influence on renal function deterioration. We were unable to control selection bias and other unmeasurable confounding factors in both the stone and non-stone subjects even using matching methods. Although we used propensity matching methods to guarantee the validity of early stage RCC patients, adequacy of early stage RCC patients as a control subject remain unclear. We were also unable to include some other established factors that influence aortic calcification and renal function, such as cigarette smoking, information on blood pressure control, medications, and presence of hydronephrosis. We could not address the direct interaction between higher ACI and having urolithiasis because of cross-sectional study. In addition, we could not address the implication of uric acid stone on ACI and renal function due to the limited number of uric acid stone patients. Many statistics were also the limitation of the present study. Therefore, additional large-scale investigations are necessary to validate the ACI impact on renal function in urolithiasis patients.

Despite these limitations, the strength of this study is that it is the first report to assess the implication of aortic calcification in urolithiasis patients. Using this non-invasive modality, we could demonstrate an independent association between ACI and renal impairment in urolithiasis patients. In addition, a possible relation of ACI and hyperuricemia through MetS is suggested. Our findings may contribute for clinicians to take intensive care and educate urolithiasis patients with severe aortic calcification to prevent renal impairment progression.

## Conclusion

In conclusion, aortic calcification and being a stone former had harmful influence on renal function. Premature vascular aging may be accelerated through MetS in stone formers. Further large-scale studies are needed to assess the clinical relevance of ACI on renal function in urolithiasis patients.

## Abbreviations

ACI: Aortic calcification
AUC: Area under the curve
CaOx: Calcium oxalate
CaP: Calcium phosphate
CIs: Confidence intervals
CKD: Chronic kidney disease
CT: Computed tomography
eGFR: Estimated glomerular filtration rate
Mets: Metabolic syndrome
ORs: Odds ratios
Q1: First quartile
Q3: Third quartile
RCC: Renal cell carcinoma
ROC: Receiver operating characteristic
